# Anticoccidial effects of *Tinospora rumphii* extract in naturally infected goats

**DOI:** 10.5455/javar.2022.i563

**Published:** 2022-01-14

**Authors:** Pearl Muyco Saladino, Elsa Amarille Gonzaga, Loveille Jun Amarille Gonzaga

**Affiliations:** 1 College of Veterinary Medicine, University of Southern Mindanao, Kabacan, Cotabato, Philippines; 2 Department of Veterinary Paraclinical Sciences, College of Veterinary Medicine, University of Southern Mindanao, Kabacan, Cotabato, Philippines; 3 Department of Chemistry, College of Science and Mathematics, University of Southern Mindanao, Kabacan, Cotabato, Philippines

**Keywords:** Coccidiostat, *Tinospora rumphii*, goats, anticoccidial activity, toltrazuril

## Abstract

**Objective:**

This study aimed to determine the anticoccidial potential of *Tinospora rumphii* ethanolic stem extract, resulting in an effective reduction in oocysts per gram counts of *Eimeria* species in goats in comparison to the commercial coccidiostat toltrazuril.

**Materials and Methods:**

Eighteen goats that were naturally infected with coccidia were identified as experimental animals. The experimental animals were grouped and were administered a single dose of *T. rumphii* extract at concentrations of 300, 600, 1,000, and 2,000 mg extract/kg body weight. The fecal samples were collected before treatment, during the first treatment, and every week thereafter for 28 days posttreatment. Fecal examination was carried out using a modified McMaster technique to determine the oocysts per gram of feces, and the mean efficacies of the extracts were calculated.

**Results:**

Stem extracts of *T. rumphii* were able to lower the oocysts per gram count than pretreatment for all concentrations, but the efficacy, in general, was still lower than toltrazuril. A significant difference existed in the efficacy of the extracts among the treatments on day 14. Administering 1,000 mg/kg extract resulted in the highest efficacy rate (95.25%) at 14 days posttreatment and was comparable to that of toltrazuril (89.89%).

**Conclusion:**

The results show that the *T. rumphii* stem extract has the potential to reduce the *Eimeria* species oocysts per gram counts of naturally infected goats.

## Introduction 

Backyard goat production in the Philippines accounts for an estimate of 3.60 million heads of goats in the inventory [1]. Goat raising is popular among rural folks since it requires simple management and low-cost production inputs compared to other livestock. Goats are vital to the economy, being kept mainly for trade, meat, and milk production. However, one of the biggest constraints in the small ruminant industry is internal parasitism. One of the principal problems in goat farming is coccidiosis, a protozoal disease caused by the Eimeria species.

Coccidiosis among young goats is commonly manifested by diarrhea, dehydration, emaciation, weakness, loss of appetite, poor weight gain, poor feed conversion, and death, causing a detrimental effect on the animal’s performance and priming for great economic loss. Acute infection is chiefly confined to kids 2–4 weeks postweaning up to 6 months of age, due to the stress of weaning, overcrowding, and unhygienic conditions, thereby exposing the goats to a large proportion of oocysts [2].

The administration of over-the-counter anticoccidial products is the common procedure of treatment. However, its high cost has limited its effective control of parasites. Moreover, a prolonged period of synthetic drug usage evidently results in it losing its strength against resistant coccidia in goats [3]. With the advent of drug resistance, alternative veterinary interventions are continuously being carried out using natural compounds to help reduce the problem.

A potential plant that can be used is Tinospora rumphii, which is synonymous with T*inospora* crispa [4]. It is a climbing shrub native to lower elevations in tropical areas. The stem of the plant is green and succulent and covered with a thin brown bark and surrounded by warty lenticels. A diverse group of pharmacologically active substances have been isolated from T. rumphii [5]. These include amino acids, glycosides, flavonoids, phenols, saponins, steroids, and terpenoids, eliciting varied bioactivities. The phytochemical profile and bioactivities of T. rumphii make it a promising source of a natural coccidiostat. Thus, this study aimed to determine the anticoccidial activity of T. rumphii stem extracts against goats naturally infected with Eimeria species.

## Materials and Methods

### Ethical approval 

The experimental procedures were approved by the University of Southern Mindanao College of Veterinary Medicine Institutional Animal Care and Use Committee MC-CVM-001552.

### Plant material and extraction

Healthy and mature stems of *T. rumphii* were selected and collected early in the morning and were validated at the University of Southern Mindanao Department of Biological Sciences. Extraction of the plant material was carried out based on the method previously described [6] with modifications and a different extracting solvent. The stems were washed thrice using tap water and then with distilled water. Stems were air-dried for 7 days prior to grinding using an electronic blender. During extraction, the powdered stems were soaked in ethanol by adding 300 ml of ethanol for every 50 gm of powder. The mixture was soaked for 48 h and filtered using Whatman filter paper. The filtrate was concentrated *in vacuo* at 40°C at 60–100 rpm to obtain the ethanolic extract and weighed to determine the concentration. The extract was stored in a clean and sterile container.

### Animals

The procedures used in the study were in accordance with the University of Southern Mindanao College of Veterinary Medicine Institutional Animal Care and Use Committee MC-CVM-001552. The methods for research and animal handling were all in compliance with Republic Act 8485, The Animal Welfare Act.

A preliminary fecal examination using the modified McMaster method [7] was carried out to determine the number of oocysts per gram (OPG) of feces and to identify which goats were infected with the Eimeria strain that will be used for the study. Identification of the Eimeria species was based on the morphometric characteristics of the oocysts and, in some cases, based on the presence or absence of a micropyle and oocyst shape [8].

Goats with an OPG of at least 875 were considered experimental animals. A total of 18 naturally coccidia-infected goats aged 3–5 months, regardless of sex and breed, but under the same management, served as the experimental animals. The management of the goats on the farm was a confined type of rearing.

The fecal samples were collected before treatment, during the first treatment, and every week thereafter for 28 days posttreatment (dpt).

### Administration of T. rumphii extract

The goats were divided into six groups (A–F) of three goats each. The experimental goats were labeled according to their treatment. The goats were weighed using a digital weighing scale to measure the live weight, which determined the volume of extract to be administered. Goats in groups A–D were given a single dose of 300, 600, 1,000, 2,000 mg extract/kg bodyweight. Those in group E were treated with a single dose of 5% toltrazuril (0.4 ml/kg bodyweight), while those in group F were untreated and served as the negative control. The goats were fasted for about 12 h to slow down gastrointestinal transit and allow better absorption of the drug administered orally [9] before the *T. rumphii* stem extract was given orally early in the morning.

### Parasitological examination

Fecal samples were collected early in the morning during feeding time at 7, 14, 21, and 28 dpt. Three to five grams of fresh fecal samples were obtained from the rectum of goats. Fecal samples were scored using a correction factor based on a scale of 1–3 with 1, normal pellets; 1.5, soft formed but the pellets did not separate; 2, soft but there was no formation of pellets; and 3, diarrheic [10]. Fecal examination was carried out using the modified McMaster technique [7] to determine the OPG of feces.

### Statistical analysis

Results are expressed as mean ± standard errors of the mean. One-way analysis of variance was used to assess the significant differences (*p* < 0.05) in mean efficacy among the groups. Furthermore, when a significant difference was found, *post-hoc* multiple mean concentration using the Tukey honestly significant difference test was used to test the consistency of the effects for treatments and controls.

## Results and Discussion

The mean OPG counts of goats in the different treatment groups are shown in Table 1. In general, there was a decrease in the OPG counts in all groups between 7 and 14 dpt except in group D. It was only at 21 dpt that group D exhibited a decrease in the OPG counts similar to all of the other groups. The OPG counts continued to decrease at 28 dpt, except for the negative control. Statistical analysis revealed that there was no significant difference in the OPG counts in goats among treatments in all periods of observation.

**Table 1.** The mean fecal oocysts per gram of naturally infected goats treated with *T. rumphii* stem extract.

Group	dpt				
	0	7	14	21	28
A (300 mg/kg)	5,925 ± 3,868	725 ± 191	875 ± 50	2,842 ± 1,109	650 ± 293
B (600 mg/kg)	2,342 ± 1,421	2,042 ± 1,702	292 ± 36	317 ± 102	142 ± 36
C (1,000 mg/kg)	1,683 ± 872	77 ± 40	408 ± 166	283 ± 8	158 ± 22
D (2,000 mg/kg)	12,267 ± 11,367	12,550 ± 12,475	20,542 ± 19,267	10,683 ± 9,859	3,117 ± 2,955
E (toltrazuril)	2,850 ± 1,815	208 ± 102	1,317 ± 1,068	1,133 ± 727	283 ± 186
F (untreated)	14,658 ± 13,741	14,142 ± 13,917	7,575 ± 7,401	317 ± 22	10,508 ± 10,000
*p*-values	0.616^*^	0.487^*^	0.523^*^	0.052^*^	0.433^*^

*: There is no significant difference at the 5% level.

The efficacy of T. rumphii stem extracts given to the goats is shown in Table 2. The results show that at 7 dpt, group C had the highest efficacy of 95.25% among the different concentrations of T. rumphii extract. Statistical analysis revealed that there was a significant difference (p < 0.05) in the efficacy of the different concentrations of T. rumphii extract used at 14 dpt. Moreover, it was observed that at 28 dpt, T. rumphii extracts had a more consistent increase in efficacy across concentrations. However, there was no significant difference observed among the six groups. Nevertheless, the highest efficacy the T. rumphii extract was able to elicit was 95.25% at a concentration of 1,000 mg/kg at 7 dpt, which was comparable to that of toltrazuril, which had an 89.89% efficacy at 7 dpt.

The efficacy of the T. rumphii extract in goats as observed in groups A, B, and C can be attributed to the anticoccidial potential of the phytochemical constituents to decrease coccidia oocyst counts in animals [11]. Saponins, tannins, and flavonoids are compounds known to have anticoccidial activity. These phytochemical compounds are present in T. rumphii stems [12].

Saponins are capable of membrane polarization, causing vacuolization and may enter the oocyst wall [13]. This action is similar to that of two conventional drugs such as praziquantel and toltrazuril, which produce severe vacuolization in all intracellular development stages, particularly in the protozoal endoplasmic reticulum, thereby interfering in the division of the protozoal nucleus, damaging the wall-forming bodies in the microgametes and also disturbing the activity of the mitochondria [14]. Saponins can directly bind to ruminal protozoa and lyse the outer membrane. However, since an oocyst wall is composed of two layers [15], saponins cannot destroy the oocyst wall but may enter the wall through the micropyle cap and damage the sporocyst [16].

Tannins may enter the inner oocyst wall and interfere with energy generation by uncoupling oxidative phosphorylation and causing a loss of intracellular components, resulting in the formation of an abnormally thickened, incomplete oocyst wall, and zygote necrosis [17]. The mode of action of tannins is quite similar to that of diclazuril, since its usage in the oocysts of both Eimeria (E.) brunetti and E. maxima resulted in the formation of an abnormally thickened, incomplete oocyst wall, and zygote necrosis [18]. Tannins also bind to glycoproteins in the cuticle of helminths and cause their death [19]. Tannins can also provide resistance through partial protective passive immunity [20].

The antioxidant properties of flavonoids may also be attributed to the anticoccidial effect of plants [21]. It was also observed that coccidia can induce host cell destruction associated with oxidative stress and lipid peroxidation [22]. The antioxidants that have the ability to neutralize reactive oxygen species (ROS) are protective due to their ROS-scavenging ability [23], reducing the severity of Eimeria infection by ameliorating the degree of intestinal lipid peroxidation. Therefore, it can be emphasized that plant extracts containing flavonoids can inhibit the development of Eimeria before oocysts are released in host feces.

Variations in the efficacy of T. rumphii extract can also result when the coccidiostat only suspends the development of the parasite at the first intracellular stage when the drug is given from the time of infection [24]. Thus, in the later stages of Eimeria species, which are developing more deeply in the intestinal tissues, the parasite becomes unaffected by the treatment given. It was shown in lambs experimentally infected with E. crandallis that diclazuril basically affects first-generation and, to a lesser extent, the late meront stages and gamonts [25]. If the meronts were destroyed by the anticoccidial treatment roughly 2 weeks after the goats had become infected, the likelihood of attaining a greater efficacy against coccidiosis might have occurred [26].

**Table 2.** The mean efficacy of *T. rumphii* stem extracts in different groups.

Group	dpt			
	7	14	21	28
A (300 mg/kg)	69.74 ± 19.23	52.68 ± 32.49a	−88.91 ± 131.21	66.70 ± 17.75
B (600 mg/kg)	−16.81 ± 106.00	65.96 ± 20.95a	50.69 ± 34.53	80.50 ± 15.25
C (1,000 mg/kg)	95.25 ± 1.19	54.47 ± 21.93a	73.35 ± 9.82	84.72 ± 6.74
D (2,000 mg/kg)	58.73 ± 33.29	−50.71 ± 9.59b	9.94 ± 8.18	79.37 ± 7.66
E (toltrazuril)	89.89 ± 4.98	66.54 ± 10.12a	61.92 ± 16.78	90.58 ± 1.98
F (untreated)	−16.11 ± 67.28	69.35 ± 11.24a	6.35 ± 83.37	49.88 ± 15.63
*p*-values	0.895^*^	0.007^*^	0.557^*^	0.277^*^

* Mean values with different letters in the same column differ significantly (*p* < 0.05).

The most common pathogenic coccidia species found in goats include E. arloingi, E. ninakohlyokimovae, E. caprovina, E. christenseni, E. faurei, and E. gilruthi. Infection is most commonly observed in kids, 2–4 weeks after weaning, and can occur in goats raised under semi-intensive and intensive management practices. The infection arises naturally when grazing, particularly when sporulated oocytes are ingested. Poor goat management practices also contribute to the infection, particularly when feed and water supplies are contaminated with goat feces or when contaminated hair coats are licked [2].

One activity that induces drug resistance is the use of year-round coccidiostats in susceptible hosts. When the number of oocysts to which animals are exposed becomes very low, the animal may fall below the threshold of exposure needed to stimulate a protective response. Therefore, coccidiostats will only be used at times of risk like parturition, shipment, weaning, or inclement weather. Toltrazuril resistance has been experimentally reported in a field isolate of ovine Eimeria species through the use of fecal oocyst reduction count as a tool to evaluate anticoccidial efficacy. Similarly, the treatment of lambs with the recommended dose of 20 mg/kg of toltrazuril (Baycox®) did not result in a significant reduction in oocyst excretion in the treated animals compared with the controls. Decreased efficacy is possibly due to the haploid stages of Eimeria species, which are immediately selected for resistance as a result of extensive use of a drug over time [24].

It is hypothesized that the variations in the efficacy of T. rumphii extract can be due to the dependence of treatment on the susceptible stages of Eimeria species present in goats. The goats in the treatment group may, in general, not be in the same stages of infection [27]. Thus, goats with coccidia in later stages of development and the short time of exposure of Eimeria species in the surface epithelium during the administration of the treatment will have lower efficacy rates. Some goats metabolize drugs so rapidly that therapeutically effective concentrations are not reached. However, further studies must be carried out regarding the influence of individual drug metabolisms on the efficacy of the extract.

## Conclusion

The result of the study shows that with the use of the stem extract of *T. rumphii*, the mean oocyst counts remained lower than the pre-treatment OPG counts except when using 2,000 mg of extract/kg bodyweight. The extract has potential *in vivo* anticoccidial activity, with a similar activity to that of the commercial coccidiostat toltrazuril at 14 dpt. 

## List of Abbreviations

dpt, days posttreatment; g, gram; HSD, honestly significant difference; kg, kilogram; mg, milligram; ml, milliliter; OPG, oocysts per gram of feces; ROS, reactive oxygen species; rpm, revolutions per minute; *T. rumphii*, *Tinospora rumphii.*
